# Long‐Term Outcomes and Follow‐Up Management in Patients With Ventricular Tachycardia Electrical Storm After Catheter Ablation

**DOI:** 10.1002/clc.70259

**Published:** 2026-01-09

**Authors:** Zhijie Song, Haizhen Guo

**Affiliations:** ^1^ Tianjin University of Traditional Chinese Medicine Tianjin China

To the Editor,

We read with great interest the article by Çöteli et al. [1] reporting long‐term outcomes of catheter ablation in patients with ventricular tachycardia‐related electrical storm (VT‐ES). The study confirms that catheter ablation is beneficial for managing ES, especially during the acute phase. Also, this high‐risk group faces substantial long‐term morbidity and mortality following the procedure. These data are valuable for guiding ES management, but several methodological limitations may undermine the reliability and generalizability of the findings and warrant further discussion.

First, the retrospective, single‐center design and small sample size of 65 patients limit the generalizability of the findings. Notably, the wide, unstratified age range (18–85 years) introduces bias because age is a well‐known risk factor for cardiovascular disease (CVD). Hence, most of the CVD studies use age stratification or restricted ranges to minimize this bias [2, 3], a safeguard that was absent here which constitutes a significant limitation.

Second, the authors identify left ventricular ejection fraction (LVEF) as the strongest predictor of post‐ablation mortality. However, the mean LVEF in this cohort was 35.3 ± 13%. It characterizes a heterogeneous group with advanced systolic dysfunction and a correspondingly high sudden‐death risk. This observation mainly mirrors the population's preexisting high‐risk profile rather than an independent ablation effect. Because reduced LVEF itself is a well‐established determinant of mortality [1], as a result, long‐term outcomes may be confounded by ongoing disease progression, thereby limiting the clinical utility of the findings. Moreover, current guidelines use 40% LVEF as a critical prognostic threshold for heart failure [4]. In a recent report, patients with ischemic heart disease, VT, and LVEF > 30% derive more long‐term benefit from early ablation than those with LVEF < 30% [5]. The impact of catheter ablation on long‐term outcome could not be properly interpreted due to not performing any subgroup analysis for either of these LVEF thresholds, nor serial postoperative LVEF measurements.

In addition, competitive risks hinder subgroup analysis between ischemic and non‐ischemic groups. Given an all‐cause mortality rate of 40%, a number of patients died from end‐stage heart failure before any recurrence of VT occurred, rendering the traditional Kaplan−Meier method inappropriate. Because it ignores competing risks and consequently misrepresents clinical reality, we recommend that Cumulative incidence functions and Gray's test are better suited to quantify the risks of recurrence and death, thereby enabling an accurate evaluation of ablation's antiarrhythmic efficacy.

To sum things up, methodological limitations warrant caution when interpreting the findings. Nevertheless, the study provides insightful evidence on the long‐term outcomes of ES patients following catheter ablation. The findings of this investigation would suggest that future investigations should prioritize prospective, multicenter cohort studies with refined subgroup analyses to inform personalized follow‐up strategies. We also propose to establish a multidisciplinary collaborative model in which pharmacovigilance for the adverse reactions of antiarrhythmic drugs is considered in prognostic assessment and follow‐up whenever applicable.

## Conflicts of Interest

The authors declare no conflicts of interest.

## Data Availability

The authors have nothing to report.
